# A Normalized Real-Life Glucose Profile After Diet-Induced Remission of Type 2 Diabetes: A Pilot Trial

**DOI:** 10.7759/cureus.23916

**Published:** 2022-04-07

**Authors:** Stefanie J Haschka, Christina Gar, Anne L Potzel, Vanessa Sacco, Stefanie Kern-Matschilles, Irina Benz, Cornelia Then, Jochen Seissler, Andreas Lechner

**Affiliations:** 1 Diabetes Research Group, Ludwig-Maximilians-Universität (LMU) Klinikum, Munich, DEU; 2 Diabetes and Endocrinology, German Center for Diabetes Research, Neuherberg, DEU; 3 Department of Internal Medicine II, Technical University of Munich School of Medicine, University Hospital Rechts der Isar, Munich, DEU; 4 Clinical Cooperation Group Type 2 Diabetes, Helmholtz Zentrum, Neuherberg, DEU

**Keywords:** diabetes mellitus type 2, metabolic syndrome, intermittently scanned continuous glucose monitoring, diabetes remission, lifestyle intervention

## Abstract

Background/objective

Type 2 diabetes related to metabolic syndrome is often partially reversible after weight loss. We conducted a pilot trial on whether complete remission to the point of a normalized real-life glucose profile, measured by continuous subcutaneous monitoring, can be achieved.

Methods

We conducted a mono-center, single-arm intervention trial between January 20, 2020, and January 12, 2021, in Munich, Germany. Ten participants had type 2 diabetes related to metabolic syndrome for a maximum of six years. They received a six-month lifestyle intervention including up to three months of a very-low-calorie formula diet, followed by stepwise food reintroduction and regular behavioral lifestyle counseling. The primary outcome was the status of glucose control at the end of the intervention. Complete remission was defined as normalization of the real-life glucose profile without glucose-lowering medication over at least five days. We measured anthropometric and biochemical parameters, body fat distribution by MRI, and insulin secretory reserve by an arginine stimulation test.

Results

Seven participants completed the trial, one reached complete remission, three achieved partial remission, and three displayed improved glucose control still in the diabetic range. A reduction of median glycosylated hemoglobin by −10 mmol/mol (−22.0 to −5.0; p = 0.016) co-occurred with weight loss of −6.4 kg (−14.2 to −3.5; p = 0.031). The insulin secretory reserve remained unchanged.

Conclusions

Complete remission of type 2 diabetes related to metabolic syndrome to the point of a normalized real-life glucose profile is possible through lifestyle intervention. Full intervention success remains challenging even with intensive counseling and support.

## Introduction

In the majority of individuals, type 2 diabetes occurs related to metabolic syndrome, i.e., in the context of overweight/obesity, ectopic fat accumulation, metabolic inflammation, and insulin resistance [1]. However, alternative pathophysiologic processes, unrelated to metabolic syndrome, can also lead to this disease [1-4].

Historically, type 2 diabetes was viewed as irreversible and progressive. However, recent research showed the opposite, at least for type 2 diabetes related to metabolic syndrome. As first demonstrated after bariatric surgery, this disease subtype can be brought into remission by substantial weight loss [5,6]. As shown later, such weight loss can also be achieved through lifestyle intervention. The Counterpoint study, for example, demonstrated that short-term caloric restriction leads to rapid metabolic improvement in subjects with type 2 diabetes related to metabolic syndrome [7]. Furthermore, the DiRECT study applied a similar approach in a routine care setting. In that trial, a 46% remission rate of type 2 diabetes related to metabolic syndrome was achieved after 12 months of a lifestyle intervention that started with a period of a very-low-calorie formula diet [8].

In the DiRECT study, remission of type 2 diabetes was defined as glycosylated hemoglobin (HbA1c) of less than 48 mmol/mol (6.5%) in the absence of glucose-lowering medication. Reaching this target of partial remission in a large percentage of study participants was a great success. Nevertheless, this remission target falls short of normalization of plasma glucose in real life and thus of a state in which glucotoxicity and beta cell stress would be minimized. Such a normalized state, however, seems necessary for the long-term maintenance of metabolic health. In contrast, partial remission to a prediabetic state is probably set up for failure and rapid reoccurrence of type 2 diabetes [9].

Currently, different devices permit obtaining a real-life glucose profile. Some transmit data continuously, while others rely on intermittent scanning. All, however, measure subcutaneous, interstitial glucose levels in short intervals. These devices are already established as valuable tools to guide insulin therapy. They provide detailed insights into glycemia that go beyond the information obtained by punctual plasma glucose measurements or HbA1c [10]. Additionally, a normal range for real-life glucose profiles has now been established through the study of healthy populations [11].

Given this level of development of continuous monitoring, real-life glucose profiling could be an ideal tool to evaluate the extent of type 2 diabetes remission. In this pilot trial, we, therefore, attempted to provide proof of concept that complete remission of type 2 diabetes related to metabolic syndrome to the point of a normalized real-life glucose profile can be achieved.

## Materials and methods

Study cohort

The monocenter lifestyle intervention study PiREM (“Pilot study for individualized REMission induction of type 2 diabetes”) was conducted at the diabetes center of the University Hospital, Ludwig-Maximilians-Universität (LMU) Munich, Germany. Participants were recruited between January and June 2020 through the local diabetes outpatient clinic as well as through advertisements on public transport and online diabetes information portals.

Inclusion and exclusion criteria

Eligible participants were 18 to 64 years old, had been diagnosed with type 2 diabetes within the previous six years, suffered from metabolic syndrome, and were motivated to participate in the lifestyle intervention. The diabetes diagnosis had to be confirmed by an HbA1c ≥ 48 mmol/mol (6.5%) plus one elevated blood glucose value (fasting glucose ≥ 7.0 mmol/l, random blood glucose ≥ 11.1 mmol/l, or 2-hour glucose in an oral glucose tolerance test ≥ 11.1 mmol/l). Furthermore, the glucose profile at baseline had to be diabetic (at least two time points with fasting sensor glucose ≥ 7 mmol/l or any sensor glucose value ≥ 11.1 mmol/l).

Exclusion criteria were autoantibodies to glutamic acid decarboxylase 65 (GAD 65), tyrosine phosphatase 2 (IA-2) or zinc transporter 8 (ZnT8) > 1.1 times the upper reference limit, fasting c-peptide < 0.27 nmol/l, recent on-record estimated glomerular filtration rate less than 30 ml/min per 1.732 m2, current or planned pregnancy within the following 12 months, scheduled surgery within the following 12 months, myocardial infarction or stroke within the previous six months, known malignancy, severe or unstable heart failure (≥ New York Heart Association class II), previous bariatric surgery, hospital admission for depression within the previous 12 months, substance abuse, or participation in another clinical trial. The current use of anti-diabetic medication, including insulin therapy, was not an exclusion criterion.

Ethics and study registration

Written informed consent was obtained from all participants and the protocol was approved by the Ethics Committee of the Ludwig-Maximilians-Universität (project number: 19-182). The study was preregistered at the German Clinical Trials Register (drks.de; study ID: DRKS00020453).

Study outcomes and design

The study was designed as a single-arm pilot trial to test the feasibility of complete diabetes remission, defined as a normalized real-life glucose profile, after lifestyle intervention. The intervention period lasted six months. In one case, it was prolonged to nine months due to study interruption and in one case, it was concluded after five months due to personal reasons.

The primary study endpoint was the degree of improvement of glucose metabolism at the post-intervention visit. Complete remission was defined as normalization of the real-life glucose profile without glucose-lowering medication over at least five days (see below for further details). In case no real-life glucose profile could be obtained, an HbA1c value of less than 39 mmol/mol (5.7%) plus a fasting plasma glucose less than 5.6 mmol/l without glucose-lowering medication were accepted as an alternative definition. However, this alternative definition of complete remission did not have to be applied. Partial remission was defined as an above-normal real-life glucose profile but an HbA1c in the prediabetic range of 39-47 mmol/mol (5.7-6.4%) without glucose-lowering medication.

Secondary endpoints were changes in body weight, BMI, waist and hip circumference, insulin resistance (calculated as homeostatic model assessment of insulin resistance (HOMA-IR)), insulin secretory reserve, whole-body, liver and pancreatic fat, systolic and diastolic blood pressure, serum lipids (triglycerides, cholesterol, high-density lipoprotein (HDL), and low-density lipoprotein (LDL) cholesterol), and changes in the prescribed medication.

The three main study visits were a screening visit, a pre-intervention visit, and a post-intervention visit that included measures of fasting plasma glucose, HbA1c, and serum lipids (triglycerides, cholesterol, HDL, and LDL cholesterol). Further, a real-life glucose profile, anthropometric measurements, MRI, and an arginine stimulation test were obtained during the pre- and post-intervention visits.

In weekly in-person visits during the first two weeks of the intervention, a study physician assessed the participants’ motivation, adherence to the diet, blood glucose profile, weight, exercise patterns, and possible side effects. After that, contacts were every other week in person or via telephone.

Intervention protocol

For participants with type 2 diabetes related to metabolic syndrome, all with a BMI ≥ 25 kg/m2, the lifestyle intervention started with a very-low-calorie formula diet (Optifast 800® or Optifast Professional®, Nestlé Health Science, Vevey, Switzerland, respectively; 815-865 kcal per day, distributed over five ratios of 163-173 kcal per day, 34-46% carbohydrates, 32-36% protein, 7-11% fat, and 5-7% fiber) for one to three months, depending on the individual progress. Only vegetables with negligible calorie content were permitted in addition to the formula diet. Participants were also advised to drink at least two liters of water or unsweetened tea per day to prevent constipation, which had been described previously in similar trials [8,12]. Further, participants were asked to monitor their plasma glucose and blood pressure. Glucose-lowering and blood pressure-lowering medications were reduced as indicated.

After a maximum period of three months, regular food was reintroduced, accompanied by regular nutrition counseling. The aim was to reach a maintenance diet that permitted further gradual weight loss or was at least isocaloric with an approximate composition of 25% carbohydrates, 25% protein/fat, 40% vegetables, and 10% fruits [13]. Besides nutrition counseling, participants were coached regarding physical activity, exercise, psychology, and behavior change. To support physical activity, pedometers were distributed. The exercise target was at least 150 minutes of moderate-intensity activity per week, preferably combined with muscle-strengthening activities [14].

Anthropometrical measurements

Height, waist circumference, and hip circumference were measured using a measuring tape to the nearest 0.5 cm. Body weight and body fat percentage were determined by bioelectrical impedance analysis (Tanita BC-418, Tanita Corporation, Tokyo, Japan). Resting systolic and diastolic blood pressures were obtained on both arms in a seated position and the mean out of two measurements on the arm with the higher systolic pressure was recorded.

MRI

Study participants were invited to participate in a whole-body MRI to determine pancreatic and hepatic fat levels via a low-fat fraction map technique (3 Tesla System, Ingenia or Achieva, Philips Health Care, Best, Netherlands). The MRI study protocol has been described in detail previously [15]. For two participants, baseline MRI data were missing due to scheduling conflicts.

Arginine stimulation test

Insulin secretory reserve was tested in an arginine stimulation test. The test protocol was adapted from Teuscher et al. [16] and Robertson [17]. Subjects with glucose-lowering medication were asked to pause their pharmacological treatment from the evening before testing. The stimulation test started after an overnight fast of at least eight hours. Fasting samples of plasma glucose and insulin were drawn, before a bolus of arginine (5 g arginine HCl, given as a 0.29 mol/l solution; B. Braun Melsungen AG, Melsungen, Germany) was injected over 60 seconds with time 0 set at the beginning of the injection. Further blood samples for insulin measurements were drawn at 2, 3, 4, 5, 6, 8, 10, and 15 minutes. The acute insulin response to arginine (AIRArg) was calculated as the mean of the three highest insulin levels obtained within five minutes after the start of arginine injection minus the pre-stimulus insulin level [18,19].

Real-life glucose profile

To obtain a real-life glucose profile, a FreeStyle® Libre 2 device (Abbott Diabetes Care, Alameda, CA, USA) was used for 2-14 days at a time. The sensor was inserted into the upper arm adipose tissue.

Interstitial glucose values were measured every minute and stored in the sensor memory every 15 minutes. For validation, occasional capillary plasma glucose measurements were implemented. Participants were advised to scan their sensor at least every eight hours to avoid losing any values stored in the sensor’s temporary memory. However, they were not supposed to use the sensor values to guide any lifestyle decisions.

A real-life glucose profile was obtained at least twice during the study, at the pre- and the post-intervention visit. An additional profile was obtained during the intervention period as needed to guide the intervention or to decide about medication.

Reference values for a normal real-life glucose profile have been published previously by Shah et al. [11]. According to this publication, we classified a profile as normal when glucose concentrations remained between 3.9 and 7.8 mmol/l at least 96% of the time. To display and analyze the real-life glucose profiles, we used the web-based diabetes management system LibreView® (Abbott Diabetes Care, Alameda, CA, USA), and the glucose values were stored in the sensor memory every 15 minutes.

Biochemical measurements

Plasma glucose was determined by the hexokinase method (Glucose HK Gen. 3, Roche Diagnostics, Mannheim, Germany), serum insulin by chemiluminescent immunoassay (DiaSorin LIAISON Systems, Saluggia, Italy), plasma HbA1c by high-performance liquid chromatography (HPLC) (VARIANT™ II TURBO HbA1c Kit, Bio-Rad Laboratories, Hercules, CA, USA), and serum blood lipids (HDL cholesterol and triglycerides) by enzymatic caloric test (Roche Diagnostics, Mannheim, Germany). LDL cholesterol was calculated by the Friedewald equation. For antibody determination, enzyme immunoassays were conducted (ZnT8: Medizym Anti-ZnT8, Medipan GmbH, Dahlewitz/Berlin, Germany; GAD65: Anti-GAD-ELISA (IgG), Euroimmun Medizinische Labordiagnostika AG, Lübeck, Germany; IA-2: Anti-IA2-ELISA (IgG), Euroimmun Medizinische Labordiagnostika AG, Lübeck, Germany). The HOMA-IR was calculated from fasting samples according to Matthews et al. [20]: HOMA-IR = glucose 0’ (mg/dl) * insulin 0* (µIU/ml)/405.

Statistical analysis

Due to the small group size, variables were treated as non-normally distributed and are presented as median (first quartile-third quartile). To compare pre- and post-intervention measurements, the Wilcoxon signed-rank test was used. Fisher’s exact test was used for the comparison of categorical variables between visits. Two-sided p-values < 0.05 were considered statistically significant. Statistical calculations were performed using the statistical software program IBM SPSS Statistics (IBM SPSS Statistics for Windows, version 25.0, IBM Corp., Armonk, NY, USA). For graphic representation, GraphPad Prism was applied (GraphPad Prism version 6.0 for Mac, GraphPad Software, La Jolla, CA, USA).

## Results

Ten participants with type 2 diabetes related to metabolic syndrome were included in this study. The participants' median age was 52 (43-56) years, they had a median BMI of 33.1 (32.1-37.7) kg/m^2^, and the median time since diagnosis was 1.5 (1.1-5.3) years (Table 1). Of these 10 participants, seven completed the study. Two dropped out due to unwillingness to follow through with the nutritional program and one because of a long-term stay abroad.

**Table 1:** Baseline characteristics of the PiREM study cohort. Values are presented as median (Q1-Q3) and frequencies are presented as n (%). T2D: type 2 diabetes; HbA1c: glycosylated hemoglobin; HOMA-IR: homeostatic model assessment of insulin resistance; LDL: low-density lipoprotein; HDL: high-density lipoprotein; MR FFM: magnetic resonance fat fraction mapping; GLP-1: glucagon-like peptide 1.

Variable	Value
Gender: male/female	5 (50%)/5 (50%)
Age (years)	52 (43-56)
Duration of T2D (years)	1.5 (1.1-5.3)
Weight (kg)	100.9 (95.3-111.6)
BMI (kg/m^2^)	33.1 (32.1-37.7)
Waist circumference (cm)	107 (104-121)
Hip circumference (cm)	111 (105.5-120)
Fasting plasma glucose (mmol/l)	9.3 (7.6-9.8)
Fasting plasma glucose (mg/dl)	167 (137-176)
Fasting insulin (pmol/l)	152.1 (121.5-320.2)
HbA1c (mmol/mol)	57.0 (52.0-63.0)
HbA1c(%)	7.4 (6.9-7.9)
HOMA-IR	8.7 (6.5-22.1)
Systolic blood pressure (mmHg)	144 (128-146)
Diastolic blood pressure (mmHg)	92 (85-98)
Triglycerides (mmol/l)	2.4 (1.5-3.6)
Triglycerides (mg/dL)	212 (135-315)
Cholesterol (mmol/l)	4.7 (4.3-5.4)
Cholesterol (mg/dL)	180 (167-209)
LDL cholesterol (mmol/l) (missing n = 1)	2.5 (2.0-3.2)
LDL cholesterol (mg/dL) (missing n = 1)	95 (76-125)
HDL cholesterol (mmol/l)	1.3 (1.0-1.5)
HDL cholesterol (mg/dL)	49 (39-57)
Body fat percentage (%)	42.1 (31.9-47.5)
Liver fat (MR FFM) (%) (missing n = 2)	20.6 (16.6-25.5)
Pancreas fat (MR FFM) (%) (missing n = 2)	1.70 (1.22-2.61)
Acute insulin response (µIU/ml)	58.4 (48.1- 100.9)
Total number of oral glucose-lowering medication	
0	5 (50%)
1	4 (40%)
≥2	1 (10%)
GLP-1 analog	1/10 (10%)
Basal insulin	1/10 (10%)

**Figure 1:**
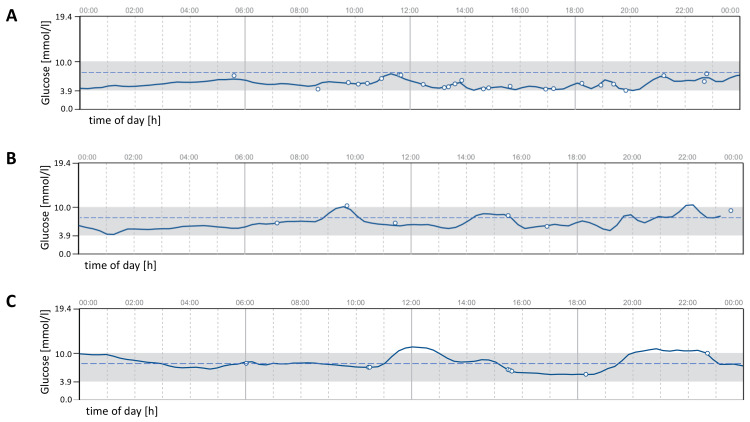
Exemplary real-life glucose profiles of participants with (A) complete and (B) partial diabetes remission or (C) an improved type 2 diabetes at the post-intervention visit. The dotted blue line represents a glucose value of 7.8 mmol/l (140 mg/dL). The graphics shown above are adaptions of the ambulatory glucose profiles generated by LibreView® (Abbott Diabetes Care, Alameda, CA, USA).

**Figure 2:**
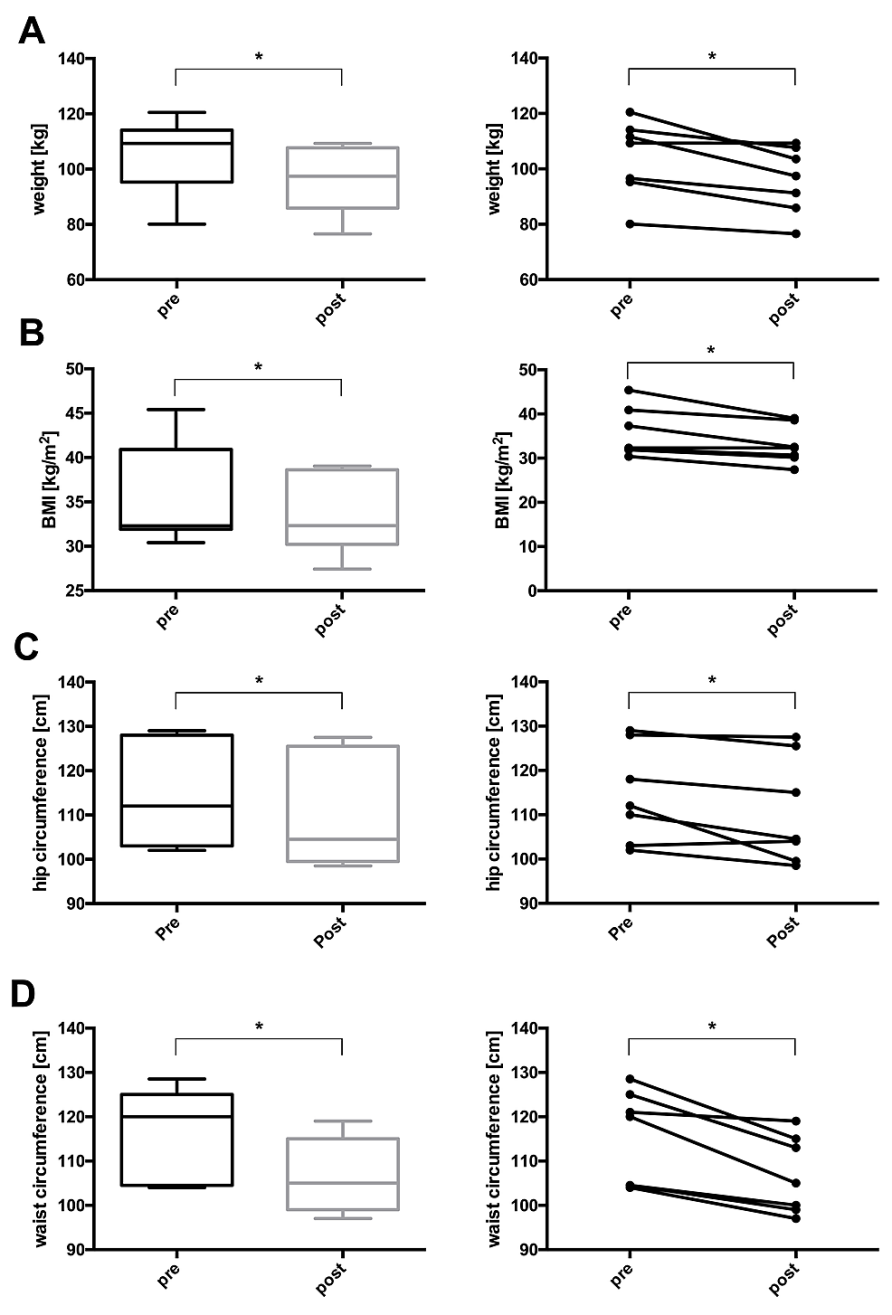
Changes in (A) weight, (B) BMI, (C) hip circumference, and (D) waist circumference before and after lifestyle intervention. Left panel (A-D): boxes represent the first quartile to the median and the median to the third quartile. Whiskers extend to the minimum and maximum values. Right panel (A-D): individual participants before and after the intervention. Note that not all y-axes start at 0. * P < 0.05.

**Figure 3:**
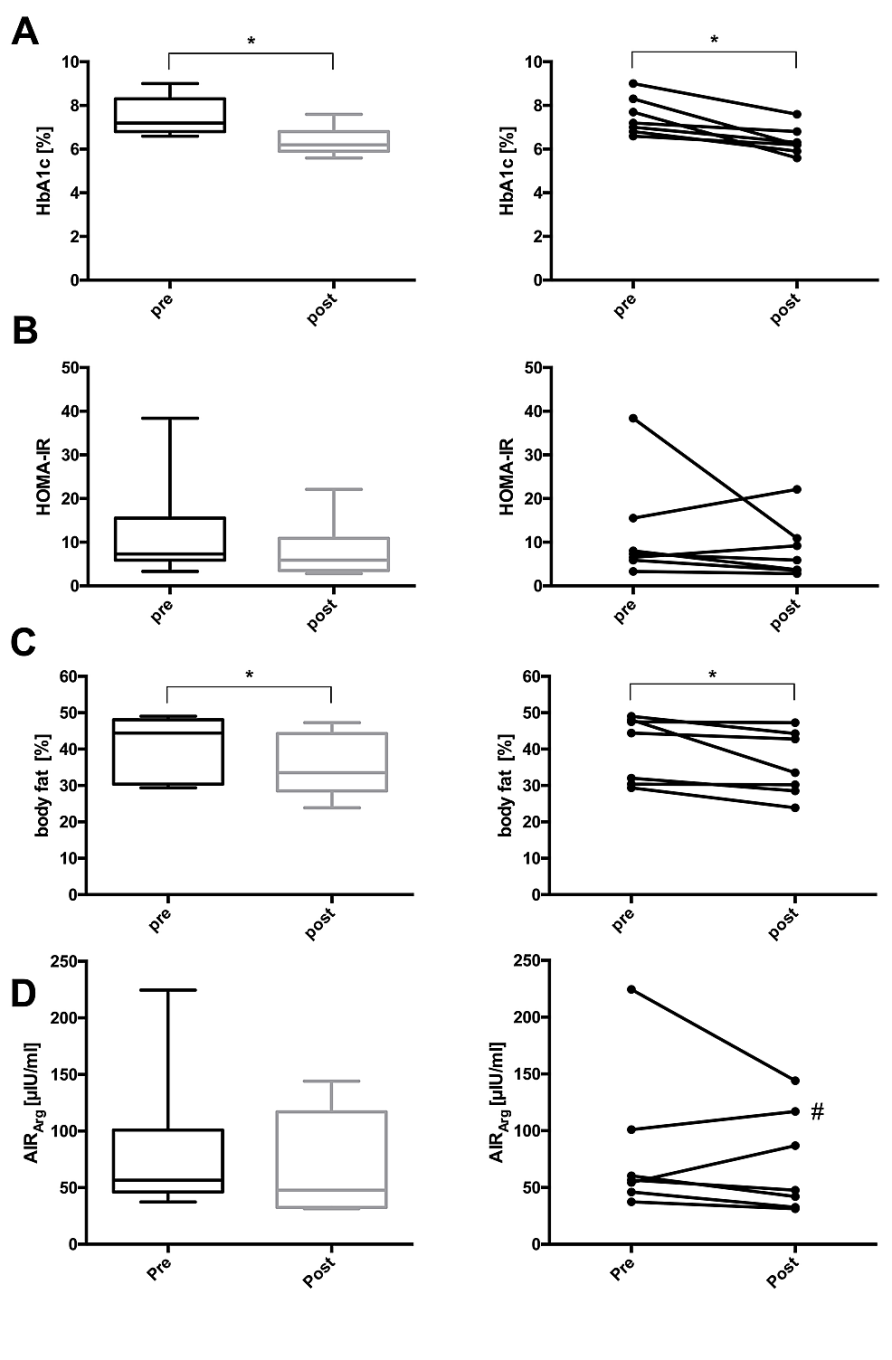
Changes in (A) HbA1c, (B) HOMA-IR, (C) body fat, and (D) acute insulin response to arginine (AIRArg) before and after lifestyle intervention. Left panel (A-D): boxes represent the first quartile to the median and the median to the third quartile. Whiskers extend to the minimum and maximum values. Right panel (A-D): individual participants before and after the intervention. * P < 0.05; # arginine stimulation test under medication (metformin). HbA1c: glycosylated hemoglobin; HOMA-IR: homeostatic model assessment of insulin resistance.

**Figure 4:**
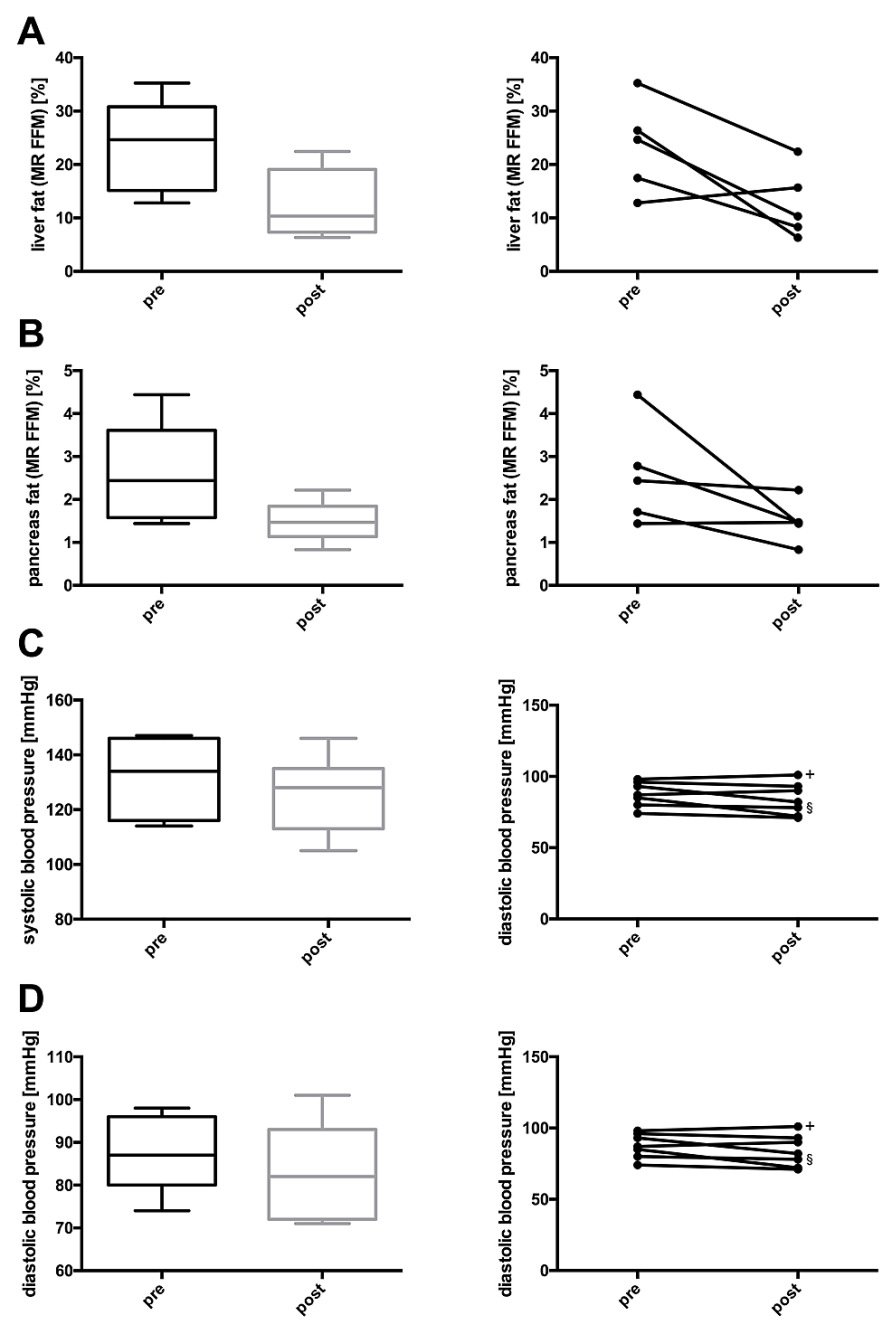
Changes in (A) liver fat, (B) pancreas fat, (C) systolic blood pressure, and (D) diastolic blood pressure before and after lifestyle intervention. Left panel (A-D): boxes represent the first quartile to the median and the median to the third quartile. Whiskers extend to the minimum and maximum values. Right panel (A-D): individual participants before and after the intervention. * P < 0.05; + discontinued blood pressure medication; § blood pressure medication cut in half.

**Figure 5:**
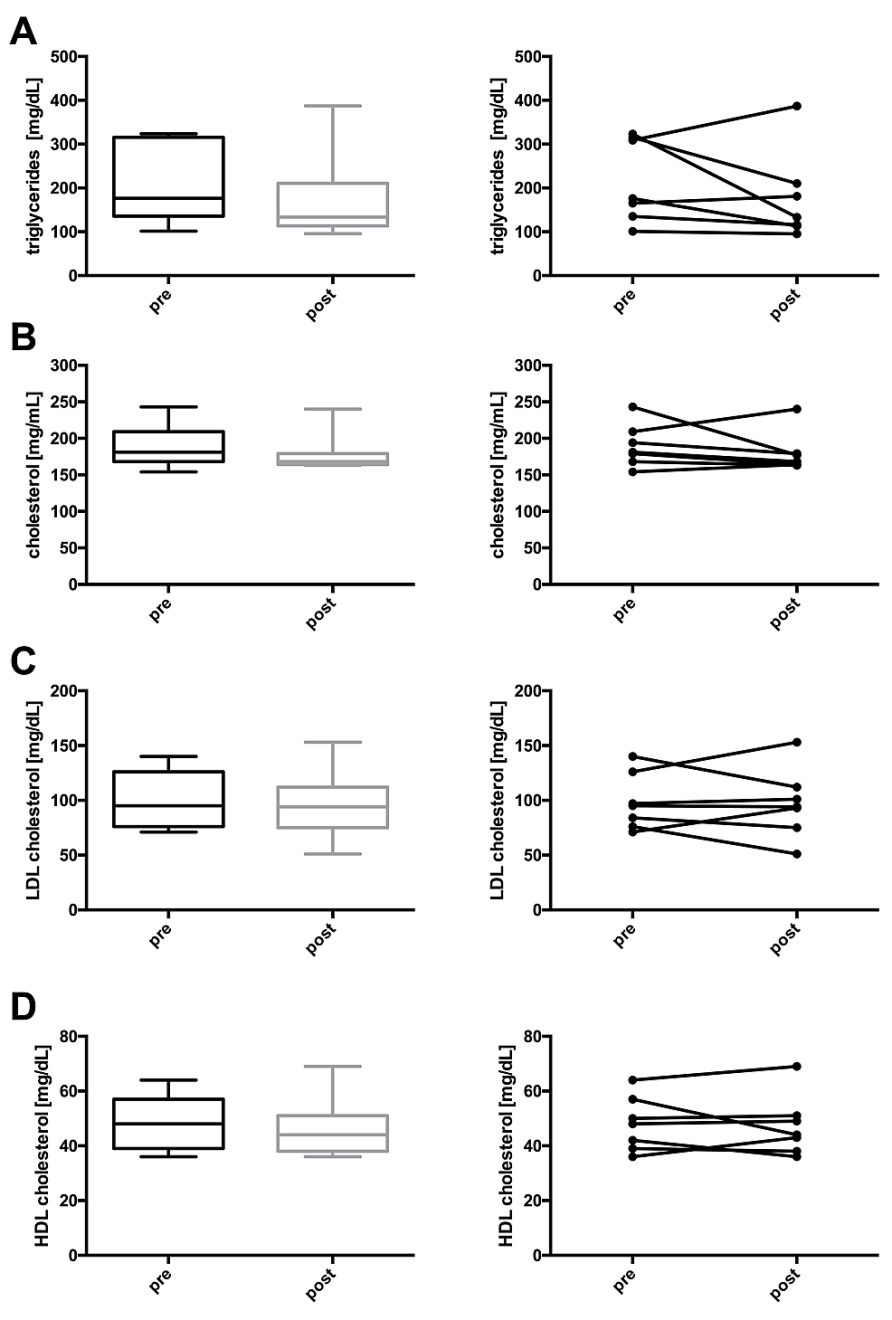
Changes in (A) triglycerides, (B) cholesterol, (C) LDL cholesterol, and (D) HDL cholesterol before and after lifestyle intervention. Left panel (A-D): boxes represent the first quartile to the median and the median to the third quartile. Whiskers extend to the minimum and maximum values. Right panel (A-D): individual participants before and after the intervention. * P < 0.05. LDL: low-density lipoprotein; HDL: high-density lipoprotein.

At the end of the study, one participant reached complete remission, three achieved partial remission, and three displayed improved glucose control still in the diabetic range (Table 2 and Figure 1). Additionally, body weight, BMI, waist circumference, hip circumference, body fat percentage, and HbA1c decreased significantly in the whole group. Fasting plasma glucose, fasting insulin, HOMA-IR, liver fat, pancreas fat, systolic and diastolic blood pressures, triglycerides, and total cholesterol depicted non-significant downward trends. LDL and HDL cholesterol, as well as the AIRArg, remained unchanged (Table 3 and Figures 2-5).

**Table 2:** Glycemic status at the post-intervention visit. Complete remission: normalized real-life glucose profile (in the normal range 96% of the time) without medication over at least five days. Partial remission: real-life glucose profile in normal range less than 96% of the time and HbA1c 39-47 mmol/mol (5.7-6.4%) without medication. Improved type 2 diabetes: improved HbA1c and unchanged or reduced glucose-lowering medication.

Glycemic status at the post-intervention visit	N	Example real-life glucose profile
Complete remission	1	Figure 1A
Partial remission	3	Figure 1B
Improved type 2 diabetes	3	Figure 1C

**Table 3:** Comparison of pre- and post-intervention measurements in the participants who completed the study. Pre- and post-intervention measurements were compared by Wilcoxon signed-rank test. Values are presented as median (Q1-Q3) and frequencies are presented as n (%). T2D: type 2 diabetes; HbA1c: glycosylated hemoglobin; HOMA-IR: homeostatic model assessment of insulin resistance; LDL: low-density lipoprotein; HDL: high-density lipoprotein; MR FFM: magnetic resonance fat fraction mapping; GLP-1: glucagon-like peptide 1.

	Pre-intervention	Post-intervention	Intra-individual difference	P-value
Gender: male/female	4 (57.1%)/3 (42.9%)	-	-	-
Age (years)	52 (47-56)	-	-	-
Duration of T2D (years)	1.4 (1.0-5.3)	-	-	-
Weight (kg) (n = 7)	109.3 (95.3-114.1)	97.4 (85.9-107.7)	−6.40 (−14.2 to −3.5)	0.031
BMI (kg/m^2^) (n = 7)	32.3 (31.9-40.9)	32.3 (30.2-38.6)	−2.30 (−4.7 to −1.4)	0.031
Hip circumference (cm) (n = 7)	112.0 (103.0-128.0)	104.5 (99.5-125.5)	−3.50 (−5.5 to −0.5)	0.047
Waist circumference (cm) (n = 7)	120.0 (104.5-125.0)	105.0 (99.0-115.0)	−7.0 (−13.5 to −4.5)	0.016
Fasting plasma glucose (mmol/l) (n = 7)	9.1 (7.2-9.7)	7.8 (7.0-8.7)	−0.9 (−1.9 to 0.6)	0.469
Fasting plasma glucose (mg/dl) (n = 7)	163.0 (130.0-174.0)	141.0 (126.0-156.0)	−17.0 (−35.0 to 11.0)	0.469
Fasting insulin (pmol/l) (n = 7)	140.3 (93.8-267.4)	110.4 (70.1-243.1)	−11.81 (−81.3 to 26.4)	0.469
HbA1c (mmol/mol) (n = 7)	55.0 (51.0-67.0)	44.0 (41.0-51.0)	−10.0 (−22.0 to −5.0)	0.016
HbA1c (%) (n = 7)	7.2 (6.8-8.3)	6.2 (5.9-6.8)	−0.9 (−2.1 to −0.4)	0.016
HOMA-IR (n = 7)	7.3 (5.9-15.5)	5.9 (3.5-10.9)	−1.4 (−4.3 to 2.7)	0.578
Systolic blood pressure (mmHg) (n = 7)	134 (116-146)	128 (113-135)	−9 (−15 to 0)	0.063
Diastolic blood pressure (mmHg) (n = 7)	87 (80-96)	82 (72-93)	−3 (−11 to 3)	0.297
Triglycerides (mmol/l) (n = 7)	2.0 (1.5-3.6)	1.5 (1.3-2.4)	−0.2 (−1.2 to 0.2)	0.297
Triglycerides (mg/dL) (n = 7)	176 (135-315)	133 (113-210)	−19 (−105 to 16)	0.297
Cholesterol (mmol/l) (n = 7)	4.7 (4.4-5.4)	4.4 (4.2-4.6)	−0.3 (−0.4 to 0.3)	0.359
Cholesterol (mg/dL) (n = 7)	181 (168-209)	168 (164-179)	−13 (−15 to 10)	0.359
LDL cholesterol (mmol/l) (n = 7)	2.5 (2.0-3.3)	2.4 (1.9-2.9)	0.0 (−0.6 to 0.6)	0.813
LDL cholesterol (mg/dL) (n = 7)	95 (76-126)	94 (75-112)	−1 (−25 to 22)	0.813
HDL cholesterol (mmol/l) (n = 7)	1.2 (1.0-1.5)	1.1 (1.0-1.3)	0.0 (−0.2 to 0.1)	1.0
HDL cholesterol (mg/dL) (n = 7)	48 (39-57)	44 (38-51)	1 (−6 to 5)	1.0
Body fat percentage (%) (n = 7)	44.4 (30.4-48.1)	33.5 (28.5-44.3)	−3.5 (−5.5 to −0.2)	0.016
Liver fat (MR FFM) (%) (n = 5)	24.66 (17.48-26.37)	10.31 (8.30-15.70)	−12.85 (−14.36 to −9.18)	0.125
Pancreas fat (MR FFM) (%) (n = 5)	2.44 (1.71-2.78)	1.47 (1.44-1.47)	−0.88 (−1.32 to −0.22)	0.125
Acute insulin response (pmol/l) (n = 7)	392.9 (320.2-700.8)	331.3 (225.9-812.6)	−61.6 (−126.9 to 111.8)	0.578
Total number of oral glucose-lowering medication				
0	3 (42.86%)	4 (57.14%)	+1	0.114
1	3 (42.86%)	2 (28.57%)	−1	
≥2	1 (14.29%)	1 (14.29%)	0	
GLP-1 analog	0/7 (0%)	0/7 (0%)	0	-
Basal insulin	1/7 (14.29%)	0/7 (0%)	−1	-

## Discussion

This pilot trial demonstrates that complete remission of type 2 diabetes related to metabolic syndrome to the point of a normalized real-life glucose profile is, in principle, possible through lifestyle intervention. However, this trial also illustrates that fully effective lifestyle change is difficult to achieve.

We consider the trial results a sufficient proof of concept for our hypothesis despite the fact that only one out of 10 participants reached the set remission target. This participant had been diagnosed with type 2 diabetes 1.4 years before study entry and had a baseline HbA1c of 51 mmol/mol (6.8 %) while taking metformin as glucose-lowering medication. Therefore, we believe that truly complete remission of established type 2 diabetes occurred in this case. Furthermore, three other trial participants reached partial remission with real-life glucose profiles in the normal range of 60-85% of the time.

As in previous studies, glycemic improvement in our trial was linked to weight loss, a reduction in hip and waist circumference, and whole-body, liver, and pancreas fat [7,21,22]. Due to the small sample size, not all of these changes were statistically significant, but the trends nevertheless appeared clear. Additionally, we observed other expected benefits of weight loss and lifestyle change, such as trends toward lower blood pressure and reduced serum triglycerides.

To avoid hyperglycemia after glucose administration, we conducted simple arginine stimulation tests to determine the insulin secretory reserve [18,19]. The median AIRArg remained stable over the study period, indicating unchanged stimulated insulin release. However, a more differentiated assessment of beta cell function may provide additional insights in future trials.

As in most lifestyle intervention trials, success in weight loss and glycemic improvement was variable between participants, despite intensive and individualized counseling [23]. This observation highlights the need for further improvements in intervention protocols for the induction of type 2 diabetes remission. We also want to reiterate that our findings and those of previous trials [7,8,21,24] only concern type 2 diabetes related to metabolic syndrome, not unrelated type 2 diabetes subtypes [1]. In those subtypes, remission by lifestyle change may be illusive or, at least, effective approaches have not been found yet.

The main limitation of our study was its small sample size. Nonetheless, we could demonstrate that complete remission of type 2 diabetes to the point of a normalized real-life glucose profile is possible in principle. Moreover, every participant completing our intervention improved his or her glycemic status and gained health benefits. Another limitation of our study was that we could not obtain baseline MRI data from two participants. Given the already small sample size, this missing data are probably responsible for the non-significant results regarding ectopic fat in the liver and pancreas despite the importance of these fat depots, which has been demonstrated in previous remission trials [7,21,22].

## Conclusions

This pilot trial proves that complete remission of type 2 diabetes related to metabolic syndrome, to the point of a normalized real-life glucose profile, is possible. The trial results thereby suggest that fat overload causes this subtype of type 2 diabetes and that weight loss can be a curative treatment for it. Larger trials should further evaluate a normalized real-life glucose profile as the remission target and the intervention protocols applied to reach it. Successful protocols could then be translated into programs for routine care.
